# Diagnostic accuracy of contrast-enhanced computed tomography and contrast-enhanced magnetic resonance imaging of small renal masses in real practice: sensitivity and specificity according to subjective radiologic interpretation

**DOI:** 10.1186/s12957-016-1017-z

**Published:** 2016-10-12

**Authors:** Jae Heon Kim, Hwa Yeon Sun, Jiyoung Hwang, Seong Sook Hong, Yong Jin Cho, Seung Whan Doo, Won Jae Yang, Yun Seob Song

**Affiliations:** 1 Department of Urology, Soonchunhyang University Hospital, Soonchunhyang University College of Medicine, 59, Daesagwan-ro, Yongsan-gu, Seoul, 140-743 Republic of Korea; 2 Department of Radiology, Soonchunhyang University Hospital, Soonchunhyang University College of Medicine, Seoul, Republic of Korea; 3 Department of Family Medicine, Soonchunhyang University Cheonan Hospital, Soonchunhyang University College of Medicine, Cheonan, Republic of Korea

**Keywords:** Renal cell carcinoma, Magnetic resonance imaging, Computed tomography

## Abstract

**Background:**

The aim of this study was to investigate the diagnostic accuracy of contrast-enhanced computed tomography (CT) and contrast-enhanced magnetic resonance imaging (MRI) of small renal masses in real practice.

**Methods:**

Contrast-enhanced CT and MRI were performed between February 2008 and February 2013 on 68 patients who had suspected small (≤4 cm) renal cell carcinoma (RCC) based on ultrasonographic measurements. CT and MRI radiographs were reviewed, and the findings of small renal masses were re-categorized into five dichotomized scales by the same two radiologists who had interpreted the original images. Receiver operating characteristics curve analysis was performed, and sensitivity and specificity were determined.

**Results:**

Among the 68 patients, 60 (88.2 %) had RCC and eight had benign disease. The diagnostic accuracy rates of contrast-enhanced CT and MRI were 79.41 and 88.23 %, respectively. Diagnostic accuracy was greater when using contrast-enhanced MRI because too many masses (67.6 %) were characterized as “4 (probably solid cancer) or 5 (definitely solid cancer).” The sensitivity of contrast-enhanced CT and MRI for predicting RCC were 79.7 and 88.1 %, respectively. The specificities of contrast-enhanced CT and MRI for predicting RCC were 44.4 and 33.3 %, respectively. Fourteen diagnoses (20.5 %) were missed or inconsistent compared with the final pathological diagnoses. One appropriate nephroureterectomy and five unnecessary percutaneous biopsies were performed for RCC. Seven unnecessary partial nephrectomies were performed for benign disease.

**Conclusions:**

Although contrast-enhanced CT and MRI showed high sensitivity for detecting small renal masses, specificity remained low.

## Background

Radiological diagnostic accuracy has evolved for patients with renal cell carcinoma (RCC) over the last two decades, such that small masses can be identified more easily [1]. These developments have led to a greater number of RCC diagnoses. The trends can be largely explained by the development of methods, such as contrast-enhanced computed tomography (CT) and contrast-enhanced magnetic resonance imaging (MRI), that enable more accurate diagnosis and more frequent diagnosis of small masses (≤4 cm, based on abdominal imaging) [2]. With an increased use of such cross-sectional imaging techniques, the majority of neoplasms (up to 80 %) are now discovered incidentally [3, 4].

CT has traditionally been regarded as the imaging modality of choice to evaluate RCC owing to its fast acquisition time and the excellent anatomic detail provided [3]. However, MRI has gained popularity for evaluating and treating RCC. MRI offers advantages, such as lack of ionizing radiation, compared to CT. More importantly, MRI can detect and classify pathologies, which makes MRI advantageous for classifying and specifying treatment outcomes, including specifying useful target therapies [3, 5].

Most RCCs are “clear-cell” RCCs, which makes this histological subtype particularly important with respect to prognosis [1, 2]. While clear-cell RCC is the most prevalent among the various categories, RCC is a heterogeneous disease that includes a large number of subtypes that differ in their histopathological features, gene expression patterns, and clinical behavior. Several studies have demonstrated the diagnostic value of contrast-enhanced CT or contrast-enhanced MRI for predicting RCC histological subtypes [6, 7].

More specifically, contrast-enhanced MRI has been described as being particularly useful for diagnosing small renal masses [8]. The most important issue with such small renal masses is judging whether they are malignant. The majority of solid masses are malignant (>80 %), but smaller masses have a greater tendency to be benign types, such as oncocytomas or angiomyolipomas. Specifically, up to 25 % of small solid renal lesions (<4 cm) are benign [9]. Notably, the distinction between a benign and a malignant mass is difficult to make for small cystic lesions (e.g., it is difficult to distinguish between multi-locular cysts and cystic RCC) [10].

Few studies have investigated the diagnostic accuracy of contrast-enhanced CT and contrast-enhanced MRI in real practice, particularly as related to treatment decisions. Thus, the overall aim of our study was to evaluate the diagnostic accuracy of the two imaging modalities and to investigate the detailed disease states of the misdiagnosed small masses.

## Methods

Contrast-enhanced CT and MRI were initially performed in 77 patients with potential small (≤4 cm) RCC, as suggested by ultrasonography. Imaging was performed at Soonchunhyang University Hospital between February 2008 and February 2013. This study was approved by the Soonchunhyang University Hospital International Review Board.

Nine of the 77 patients refused surgical treatment. Those undergoing cyto-reductive nephrectomies and those with documented metastatic disease prior to surgical intervention were excluded. Such patients were excluded because most were known to have conventional RCC. Thus, 68 patients were available for the data analysis. Contrast-enhanced CT and MRI were performed on small masses (≤4 cm in diameter), as detected by conventional CT or abdominal ultrasonography.

The small renal masses were routinely evaluated using contrast-enhanced CT and contrast-enhanced MRI in corticomedullary, venogenic, and nephrographic phases. Two experienced urologic radiologists (HSS and JYH), who made the original interpretations, conducted a retrospective review of the contrast-enhanced CT and MRI scans.

Contrast-enhanced computed tomography was performed in unenhanced, corticomedullary, and nephrographic phases, using 64-channel scanners (Sensation 64; Siemens Medical Solutions, Erlangen, Germany). Unenhanced images were obtained, and then, an intravenous contrast agent (Omnipaque 320 [iohexol, GE Medical System Milwaukee, WI, USA; or Iomeron 350 [iomeprol], Bracco, Milano, Italy) was injected using a power injector at a dose of 2 mL/kg of body weight and a rate of 3.0 mL/s up to a maximum of 150 mL. The scan delay of corticomedullary phase scanning was determined by an automatic bolus triggering technique of MDCTs; scanning started when the CT number of a region of interest (ROI) in the abdominal aorta reached 100 HU. The scan delay for nephrographic phase scanning was 180 s. The scanning parameters were as follows: X-ray tube voltage, 120 kV; tube current, 100–250 mA, which was determined by an automatic dose modulation technique; and slice thickness/reconstruction interval, 5 mm/5 mm for unenhanced and 3 mm/3 mm for corticomedullary phase scanning and early excretory phase scanning.

MR imaging was performed using a 3.0-T unit (Discovery MR750w; GEHealthcare, Milwaukee, WI, USA) with a phased-array coil. After localizer images were acquired, the following sequences were obtained: (a) coronal T2-weighted single-shot fast spin echo without fat saturation (repetition time msec/echo time msec, 1500/90; 90° flip angle; bandwidth, ±83 kHz; field of view, 40 cm; section thickness, 5 mm; gap, 0.5 mm; 320 × 288 matrix); (b) axial T2-weighted single-shot fast spin echo without fat saturation (repetition time msec/echo time msec, 1500/80; 90° flip angle; bandwidth, ±83 kHz; field of view, 34 cm; section thickness, 5 mm; gap, 0.5 mm; 384 × 256 matrix); (c) axial volumetric 3D fat fraction sequence, called iterative decomposition of water and fat with echo asymmetry and least square estimation (IDEAL-IQ; GE healthcare) (repetition time of 6.6 msec and six different echo times that ranged from 1.6 to 9.8 msec; 15° flip angle; bandwidth, ±142 kHz; field of view, 34 cm; section thickness, 4.6 mm; gap, 2.3 mm; 272 × 224 matrix); and (d) three-dimensional fat-saturated T1-weighted GRE images (5.9/1.1; 15° flip angle; section thickness, 4.6 mm; bandwidth, ±142 kHz; field of view, 34 cm; 320 × 224 matrix) obtained before and after administration of an intravenous bolus of 0.1 mmol/kg of gadoteridol (Prohance; Bracco, Milano, Italy) at a rate of 1.5–2.0 mL/s, and followed by a 20-mL saline flush. Contrast agent-enhanced images were acquired in the corticomedullary and nephrographic phases using an automatic bolus triggering technique. The nephrographic phase was initiated 30–40 s after the corticomedullary phase.

An apparent diffusion coefficient (ADC) map was obtained at each slice position. The ADC was measured in an approximately 1-cm region of interest within the normal renal parenchyma. ADC values in normal renal parenchyma ranged from 1.72 × 10^−3^ mm^2^ s^−1^ to 2.65 × 10^−3^ mm^2^ s^−1^.

For visual assessment and to provide quantitative diagnostic criteria with the abovementioned techniques, a 5-point scale was used: 1 indicates definitely fluid or definitely not cancer: a benign simple cyst or water density without enhancement; 2 indicates probably fluid or probably not cancer: a benign cyst of thin septa with a few hairline septa. For solid lesions, uniformly high-density cysts with clear margins and without enhancement can be present; 3 represents an indeterminate risk of cancer: measurable enhanced wall or septa with irregular thickening and smooth wall; 4 indicates probable cystic or solid cancer: irregular marginated cystic masses with enhanced soft-tissue components; and finally, 5 indicates definite cystic solid cancer: clear cystic or solid malignant masses with or without calcification and with irregular vascularity. There is a prominent gap in the enhancement pattern between the mass and the cortex during the corticomedullary phase.

The interpretations were re-categorized using the 5-point scale (“1 (definitely fluid or definitely not cancer), 2 (probably fluid or probably not cancer), 3 (indeterminate risk of cancer), 4 (probably solid cancer), or 5 (definitely solid cancer)”) by the two radiologists and by an attending urologic oncologist who was blinded to the radiological images and to the final pathological findings. Ratings of 1 to 2 were labeled “non-cancer,” whereas those of 3 to 5 were labeled “cancer.”

Diagnostic accuracy was defined by whether masses were categorized as “4 (probably solid RCC) or 5 (definitely solid RCC)” or “3 (indeterminate RCC)” tumors. The decision to use these categories was supported by the tendency of urologists to most commonly label specific diseases as either “4 (probably solid RCC) and 5 (definitely solid RCC)” or “3 (indeterminate RCC)” tumors.

Accuracy including sensitivity, specificity, and positive and negative predictive values was analyzed using receiver operating characteristic (ROC) curve. STATA version 14 software (Stata Corp LP, College Station, TX, USA) was used for statistical analysis, and graphs were generated by MedCalc—version 13.0.4. Significant differences were defined by *P* < 0.05.

## Results

Among the 68 patients, 60 (88.2 %) had RCC and eight had benign disease (Table 1). Among those with RCC, 51 (75.0 %) had clear-cell RCC and nine (13.2 %) had papillary or choromophobe RCC. Among the patients with benign lesions, four (5.88 %) had oncocytoma and three had angiomyolipoma, multi-locular cysts, or papillary tubule-adenoma.

**Table 1:** Characteristics of the study subjects (*n* = 68)

Age	63.1
Male	47 (69.11)
Female	21 (30.88)
Method of pathologic confirmation	
Percutaneous biopsy	4 (5.88)
Partial nephrectomy	42 (61.7)
Radical nephrectomy	20 (29.4)
RCC	60 (88.2)
Clear-cell RCC	51 (75.0)
Papillary or chromophobe RCC	9 (13.2)
Metastatic adenocarcinoma	1 (1.47)
Benign mass	7 (10.2)
Oncocytoma	4 (5.88)
Angiomyolipoma	1 (1.47)
Multi-locular cyst	1 (1.47)
Tubulo-papillary adenoma	1 (1.47)

*n* number, *RCC* renal cell cancer

The mean patient age was 63.1 years. Forty-seven patients were male and 21 were female. All RCCs were in the T1a stage. All were solid or cystic masses, with no renal capsule or vessel involvement.

**Fig. 1:**
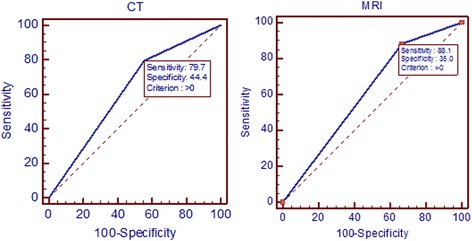
Sensitivities and specificities of dynamic computed tomography and dynamic magnetic resonance imaging for prediction of the final diagnosis

The diagnostic accuracies of contrast-enhanced CT and MRI were 79.41 and 88.23 %, respectively (Table 2). A tendency was observed for masses to be diagnosed as “4 (probably solid RCC) and 5 (definitely solid RCC)” at a higher rate by contrast-enhanced MRI than by contrast-enhanced CT. The contrast-enhanced MRI characterization rate of the “4 (probably solid RCC) and 5 (definitely solid RCC)” state was 67.6 % (Table 2). The sensitivities for contrast-enhanced CT and MRI for the prediction of RCC were 79.7 and 88.1 %, respectively. The specificities for contrast-enhanced CT and MRI were 44.4 and 33.3 %, respectively (Fig. 1).

**Table 2:** Diagnostic accuracies of CT and MRI (*n* = 68)

Radiologic interpretation	“4 (probably solid RCC) and 5 (definitely solid RCC)”		“3 (indeterminate RCC)”		Total
CT	33 (48.5)		21 (30.8)		54 (79.41)
MRI	46 (67.6)		14 (20.58)		60 (88.23)
Radiologic interpretation	Sensitivity	Specificity	AUC	SE^a^	95 % CI^b^
CT	79.7	44.4	0.621	0.0917	0.495 to 0.736
MRI	88.1	33.3	0.607	0.0860	0.481 to 0.724

*n* number, *CT* computed tomography, *MRI* magnetic resonance imaging

^a^Binomia exact

^b^Binomial exact

The diagnostic advantage of contrast-enhanced MRI is described in detail in Table 3. The diagnoses of 14 patients were upgraded from “3 (indeterminate RCC)” to “4 (probably solid RCC) or 5 (definitely solid RCC)” in five patients (7.35 %) (Table 3).

**Table 3:** Advantage of diagnostic upgrading by MRI (*n* = 14)

Final diagnosis	*N* (% for total *N*)	CT interpretation	MRI interpretation	Treatment
Clear RCC	13 (19.11)			
	8 (11.76)	“3 (indeterminate RCC)”	“4 (probably solid RCC) or 5 (definitely solid RCC)”	Partial Nx
	5 (7.35)	“3 (indeterminate RCC)”	“4 (probably solid RCC) or 5 (definitely solid RCC)”	Partial Nx
Papillary RCC	1 (1.47)	“3 (indeterminate RCC)”	“4 (probably solid RCC) or 5 (definitely solid RCC)”	Partial Nx
Total	14 (20.5)			

*N* number, *CT* computed tomography, *MRI* magnetic resonance imaging, *RCC* renal cell cancer, *Nx* nephrectomy

Fourteen cases were missed or inconsistently diagnosed, compared with the final pathological diagnoses (20.5 % of all cases) (Table 4). One appropriate nephroureterectomy and two unnecessary percutaneous biopsies were performed for clear RCCs. Three unnecessary percutaneous biopsies were performed for papillary or choromophobe RCCs. Seven unnecessary partial nephrectomies were performed in patients with benign disease. Those seven cases included three cases of oncocytoma, one case of acute myeloid leukemia, one case of metastatic adenocarcinoma, and one multi-locular cyst.

**Table 4:** Diagnoses missed by CT and MRI (*n* = 14)

Final diagnosis	Clinical impression	*N* (% for total *N*)		Shape of mass	CT interpretation	MRI interpretation	Treatment
Clear RCC	TCC	3 (4.41)	Case #1	Solid	“3 (indeterminate RCC),” “4 (probably solid TCC)”	“3 (indeterminate RCC),” “4 (probably solid TCC)”	Nephroureterectomy
	AML		Case #2	Solid	“4 (probably solid AML)”	“3 (indeterminate RCC),” “4 (probably solid AML)”	Percutaneous biopsy and partial Nx
	Complicated cyst		Case #3	Cystic	“2 (probably fluid)”	“2 (probably fluid),” “3 (indeterminate RCC)”	Percutaneous biopsy and partial Nx
Papillary or chromophobe RCC	Complicated cyst	3 (4.41)	Case #1	Cystic	“2 (probably fluid)”	“2 (probably fluid),” “3 (indeterminate RCC)”	Percutaneous biopsy and partial Nx
	Complicated cyst		Case #2	Cystic	“2 (probably fluid)”	“2 (probably fluid),” “3 (indeterminate RCC)”	Percutaneous biopsy and partial Nx
	AML		Case #3	Cystic	“3 (indeterminate RCC)”	“4 (probably solid RCC)”	Percutaneous biopsy and partial Nx
Angiomyolipoma	RCC	1 (1.47)	Case #1	Solid	“3 (indeterminate RCC)”	“4 (probably solid RCC)"	Partial Nx
Oncocytoma	RCC	4 (5.88)	Case #1	Solid	“3 (indeterminate RCC)”	“4 (probably solid RCC)”	Partial Nx
	RCC		Case #2	Solid	“3 (indeterminate RCC)”	“4 (probably solid RCC)”	Partial Nx
	RCC		Case #3	Solid	“4 (probably solid AML)”	“3 (indeterminate RCC)”	Partial Nx
	RCC		Case #4	Solid	“4 (probably solid AML)”	“3 (indeterminate RCC)”	Partial Nx
Adenocarcinoma	RCC	1 (1.47)	Case #1	Cystic	“3 (indeterminate RCC)”	“3 (indeterminate RCC)”	Partial Nx
Multi-locular cyst	RCC	1 (1.47)	Case #1	Cystic	“4 (probably solid RCC)”	“3 (indeterminate RCC)”	Partial Nx
Total		14 (20.58 %)					

*N* number, *CT* computed tomography, *MRI* magnetic resonance imaging, *RCC* renal cell cancer, *TCC* transitional cell carcinoma, *AML* angiolipoma, *Nx* nephrectomy

## Discussion

The role of imaging studies when deciding the treatment modality for renal masses is extremely important. The subjective visual impression by a radiologist has a known critical role in differentiating a simple cyst from a solid mass [11, 12]. Moreover, the precision of this subjective impression for detecting a mass is improved by the use of contrast-enhanced CT or contrast-enhanced MRI [13].

However, the real role of the subjective impression based on contrast-enhanced CT or contrast-enhanced MRI has not been fully evaluated, particularly when detecting small renal masses. Most studies have demonstrated a high accuracy of contrast-enhanced CT and MRI through retrospective analyses. However, most urologists make clinical decisions based on the subjective impression of a radiologist.

The greatest pitfalls of clinical studies regarding the diagnostic accuracy of small renal masses include the disparity of viewpoints between urologists and radiologists. Radiologists generally focus on the accuracy of radiologic imaging according to their final pathological reports, and urologists generally only focus on the success rate of surgery. Hence, there have been few studies on the real value of radiologic imaging in the establishment of a treatment plan or regarding treatment with surgery or other procedures without considering the real diagnosis. The current study, although handicapped by small inclusion numbers, addresses this issue.

Diagnostic and staging accuracy for renal masses has been investigated for multiple imaging modalities, including ultrasound sonography, CT, and MRI [13]. Although some reports have investigated the diagnostic efficiency of contrast-enhanced MRI for the detection of small clear-cell RCC [8], small renal masses have often been neglected. Few studies have assessed the correlation between a diagnosis of small renal mass and treatment strategy [14, 15]. This is the first report that attempts to clarify the clinical value of contrast-enhanced CT and contrast-enhanced MRI in establishing a treatment strategy for small renal masses.

CT has been the traditional imaging modality of choice for detecting RCC and for RCC staging work-up. CT provides excellent anatomical detail, allowing for complex three-dimensional reconstruction of a renal tumor and the vascular anatomy [3, 5]. However, a pitfall of CT when evaluating a renal mass is that it cannot detect small renal masses [16, 17]. An artificial alteration for CT when assessing renal lesions, particularly small lesions, is a “partial volume artifact,” which often results in an incorrect diagnosis [18].

MRI has grown in popularity owing to its advantages in the histological characterization of masses [19, 20]. MRI offers the advantages of excellent non-ionizing radiation exposure and exquisite tissue characterization compared with CT. In particular, MRI allows for excellent characterization of cystic and solid masses owing to its ability to detect hemorrhage, intracellular fat, and intra-cystic architecture using various MRI techniques including diffusion-weighted images or arterial spin labeling or MR spectroscopy [3]. CT has proven limitations for detecting RCC [10, 16, 17].

Use of MRI for renal imaging has traditionally been limited to cases in which diagnosis by ultrasonography or CT was inconclusive and cases in which the presence or absence of tumor thrombi was being investigated [21]. MRI has replaced venacavography as the gold standard when evaluating extensions of renal tumors into the inferior vena cava. MRI is considered superior to spiral CT for this application, with a sensitivity of 100 % and a specificity of approximately 90 % [22].

The diagnostic abilities of CT and MRI to predict the pathologic diagnosis have been evaluated. Most studies have focused on distinguishing RCC from benign entities in clear cells. This is an important issue, as clear-cell RCC has different characteristics than do other types of RCC and the prognosis for clear-cell RCC is usually worse than for other types of RCC.

Contrast-enhanced MRI has been used to detect small renal masses and to predict RCC pathological subtypes [8]. Diverse MRI techniques, such as diffusion-weighted imaging, have enhanced the role of MRI in detecting RCC among small renal masses [10]. However, these techniques require a ROI, which can vary between and within observers, based on the size and position of the ROI [23]. However, those reports are based on thorough retrospective review and analysis of images, and this is not a realistic situation in real practice. Hence, we focused on the role of subjective interpretation of contrast-enhanced CT and contrast-enhanced MRI, which can help clinicians make prompt decisions.

The CT findings including enhancement pattern has shown significant association with histological subtypes of renal cell cancer [19, 20, 24, 25]. In CT findings, clear-cell RCC tends to contain suspected necrosis with heterogenous enhancement pattern compared with chromophobe and papillary RCCs [19, 25]. The imaging characteristics of CT predict the clear-cell RCC with an accuracy of 72 %, but shows lower accuracy in small renal tumors ≤5 cm [20].

In our analysis, CT or MRI findings did not reveal different necrosis patterns. Because most tumors were small with a round pattern, it was difficult to analyze pattern nodularity, tumor shape, or round margins.

RCC subtyping using contrast-enhanced MRI has been investigated with excellent results [6–8]. We found previously that signal intensity changes during the corticomedullary phase are the most effective means of distinguishing small clear-cell from papillary cell RCC [8].

Similar to small solid renal masses, small renal cysts also have complexities with regard to RCC detection [26]. A popular CT-based classification system to determine the malignant potential of a cystic kidney lesion is the Bosniak classification system, which classifies cysts from benign or simple cyst to malignant RCCs [27]. Clinically challenging complex cystic lesions are Bosniak IIF and Bosniak III, which could reveal malignancy up to 50 % [28]. CT has traditionally been regarded as the modality of choice for evaluating renal cysts [27, 29]. Israel et al. [27] reported the superiority of MRI to CT regarding sensitivity in their retrospective review of 69 cystic renal lesions. Although they reported the greater sensitivity of MRI in complicated cystic lesions, they did not investigate about the specificity of CT or MRI.

Our study revealed that the sensitivities of subjective impressions for contrast-enhanced CT and MRI to predict RCC were 79.7 and 88.1 %, respectively, and the specificities of contrast-enhanced CT and MRI for predicting RCC were 44.4 and 33.3 %, respectively. These results indicate that the accuracy of subjective impression for contrast-enhanced CT and MRI is not as high as previously reported, which could result in either over- or under-treatment. In our study, two cases of papillary RCC were misdiagnosed as complicated cysts by both contrast-enhanced CT and MRI, suggesting a challenge to reach a clear diagnosis for small renal cystic masses.

In our study, although the sensitivity of diagnostic accuracy for contrast-enhanced MRI was higher than that for contrast-enhanced CT, specificity was low for both imaging modalities. This indicates that a serious RCC lesion could be missed by both imaging modalities. In addition, more aggressive treatment modalities were suggested for benign masses in some cases. Three unnecessary percutaneous biopsies and seven unnecessary partial nephrectomies were performed. Thus, further studies are needed to investigate the real role of contrast-enhanced CT and MRI in predicting the pathologic diagnosis of such small renal masses. Advanced contrast-enhanced MRI techniques may be useful for achieving better diagnostic accuracy.

There are several limitations to our study. First, the number of study subjects is small. However, other reports regarding this issue of small renal masses also do not include a large number of subjects [8–10]. Second, owing to the treatment methods of this hospital, we could not consider other minimally invasive treatments, such as radiofrequency or cryoablation [30, 31].

## Conclusions

Although contrast-enhanced CT and contrast-enhanced MRI offer high sensitivity for a precise diagnosis of small renal masses, the specificity of these techniques is low. A closer examination of these issues by urologic oncologists and radiologists is necessary to overcome the low specificity.

## Abbreviations

CT: Computed tomography
MRI: Magnetic resonance imaging
RCC: Renal cell carcinoma
